# Lehre des Kopf-Hals-Ultraschalls: Übersicht aktueller Lehrformate und Prüfungsstrukturen sowie ihr möglicher Einfluss auf die Weiterentwicklung des Curriculums

**DOI:** 10.1007/s00106-025-01712-2

**Published:** 2025-12-18

**Authors:** Alisa Schramm, Matthias Tisch, Christian Offergeld

**Affiliations:** 1 Klinik für Hals-Nasen-Ohren-Heilkunde, Kopf- und Halschirurgie, Bundeswehrkrankenhaus Ulm, Oberer Eselsberg 40, 89081 Ulm, Deutschland; 2 Klinik für Hals‑, Nasen- und Ohrenheilkunde, Universitätsklinikum Freiburg, Freiburg, Deutschland

**Keywords:** Prüfungsformate, Zertifizierung, Qualitätssicherung im Gesundheitswesen, Digitalisierung, Medizinische Ausbildung, Credentialing, Certification, Health care quality assurance, Educational assessment, Medical education

## Abstract

**Hintergrund:**

Die Sonographie stellt ein unverzichtbares Diagnostikinstrument in den Kopf-Hals-Fachgebieten dar. Trotz allen technischen Fortschritts bleibt die Untersuchungsqualität in beträchtlichem Maße von der Qualifikation des Untersuchers abhängig, sodass hohe Anforderungen an den Ausbildungsstandard gestellt werden müssen. Obwohl die Inhalte der Ultraschallkurse im Kopf-Hals-Bereich (UK-KH) homogen sind, unterscheiden sich die Kurse bezüglich der Formate und Prüfungsmodalitäten.

**Ziel der Arbeit:**

Die vorliegende Arbeit gibt einen vergleichenden Überblick über die 2025 in Deutschland verfügbaren UK-KH mit und ohne Beteiligung der Schilddrüse. Besonderes Augenmerk liegt dabei auf den gewählten Lehrformaten (Präsenz, digital, hybrid) sowie den angewandten Prüfungsformaten zur Kompetenzüberprüfung.

**Material und Methoden:**

Im ersten Schritt erfolgte die Bestandsaufnahme der in Deutschland angebotenen UK-KH und die Kursanalyse über frei verfügbare Flyer. Zum Abgleich und der Ergänzung von kursspezifischen Informationen wurden anschließend Telefoninterviews anhand eines Leitfadens geführt.

**Ergebnisse:**

Es wurden 12 Kursanbieter identifiziert, wobei die Mehrheit (*n* = 10) Präsenzkurse anbietet. Ferner existieren ein vollständig digitales, ein etabliertes hybrides und ein in der Erprobungsphase befindliches hybrides Ausbildungskonzept. Eingesetzte Prüfungsformate reichen von praktischen Prüfungen über Multiple-Choice-Tests. Teilweise wurde auf eine Leistungsüberprüfung verzichtet.

**Schlussfolgerung:**

Während digitale Lehr- und Prüfungsformen als zunehmend relevant eingeschätzt werden, erfolgt ihre systematische Integration in UK-KH bislang nur vereinzelt. Um die Wirksamkeit verschiedener Formate verlässlich beurteilen und zukünftige Innovationen gezielt fördern zu können, sind weitere Studien und standardisierte Verfahren zur Erfassung von Untersuchungsqualität und Kompetenzerwerb erforderlich.

## Abstract (en)

**Background:**

Ultrasound is an indispensable diagnostic tool in head and neck specialties. Despite technological advances, examination quality remains highly dependent on the examiner’s qualification, thus placing substantial demands on training standards. Although the content of head and neck ultrasound courses in Germany (UK-KH) is homogeneous, the course formats and assessment modalities vary.

**Objective:**

This study provides a comparative overview of the UK-KH available in Germany in 2025, both with and without thyroid-focused content. Particular emphasis is placed on the chosen teaching formats (in-person, digital, hybrid) and the assessment methods used to evaluate competency.

**Materials and methods:**

First, all UK-KH offered in Germany were identified and systematically analyzed using publicly available course flyers. To verify and supplement course-specific information, structured telephone interviews based on a predefined protocol were subsequently conducted.

**Results:**

Twelve course providers were identified, with the majority (*n* = 10) offering in-person courses. In addition, one fully digital, one established hybrid, and one hybrid concept currently in the testing phase were identified. Applied assessment formats included practical examinations and multiple-choice tests as well as instances in which no performance assessment was undertaken.

**Conclusion:**

Although digital teaching and assessment methods are considered increasingly relevant, their systematic integration into UK-KH remains limited. To reliably evaluate the effectiveness of different formats and to promote upcoming innovation, further studies and standardized procedures for measuring examination quality and skill acquisition are required.

Wie lehren wir Kopf-Hals-Ultraschall? Der Ultraschall hat sich in den vergangenen Jahren als unverzichtbares Diagnostikinstrument in der Hals-Nasen-Ohren-Heilkunde und der Mund-Kiefer-Gesichts-Chirurgie etabliert. Die vorliegende Arbeit bietet einen vergleichenden Überblick der für das Jahr 2025 verfügbaren Kopf-Hals-Ultraschallkurse in Deutschland. Ziel ist es, bestehende Konzepte übersichtlich und systematisch darzustellen und Entwicklungspotenziale aufzuzeigen. Der Fokus liegt dabei auf den Lehrformaten und Prüfungsstrukturen.

## Diagnostische Qualität

Trotz aller technologischen Fortschritte – von höherer Bildauflösung über kompakte, breit einsetzbare Geräte bis hin zur Reduktion des Untersuchungsaufwands und zur verbesserten Verfügbarkeit – bleibt die diagnostische Qualität jedoch maßgeblich von der Erfahrung und Qualifikation des Untersuchers abhängig. Diese gewährleistet eine verlässliche Befundung und kommt dadurch letztlich in besonderem Maße dem Patienten zugute. Daher ist die strukturierte, praxisnahe Ausbildung eine wichtige Grundlage für die hohe Versorgungsqualität. Wenngleich reine Fallzahlen allein noch keinen versierten Untersucher garantieren, sind von den Landesärztekammern Mindestanforderungen definiert, die sich an den Empfehlungen der Muster-Weiterbildungsordnung von 2018, in der aktualisierten Fassung vom 14.06.2024, orientieren. Diese sieht bspw. für die Weiterbildung im Gebiet Hals-Nasen-Ohren-Heilkunde 400 sonographische Untersuchungen, davon 100 der Gesichts- und Halsweichteile, 100 der Nasennebenhöhlen und 200 Doppler‑/Duplexsonographien der extrakraniellen hirnversorgenden Gefäße vor [1]. Die richtungsweisende Untersuchung der Schilddrüse wird zwar ebenfalls als Bestandteil genannt, jedoch ohne konkrete Richtzahl.

## Nachweis der fachlichen Befähigung

Um jungen Kollegen das Erlernen des Kopf-Hals-Ultraschalls in einem dezidierten Lernumfeld zu ermöglichen, gibt es in Deutschland zahlreiche Kursangebote, die über die Jahre eine zunehmende Professionalisierung erfuhren. Dazu trugen die Kassenärztliche Vereinigung (KV) und Fachgesellschaften wie die Deutsche Gesellschaft für Ultraschall in der Medizin (DEGUM) bei, indem klare Rahmenbedingungen zu Kursumfang, Inhalten und praktischen Anteilen vorgegeben wurden. Die bundesweite Vereinheitlichung des Lehrangebots ist ferner darauf zurückzuführen, dass der KV-konforme Nachweis der fachlichen Befähigung (im Rahmen der Weiterbildungsordnung, einer ständigen Tätigkeit oder durch Ultraschallkurse) Voraussetzung für die Abrechnung sonographischer Leistungen ist. So wird einerseits die flächendeckende Qualifikation und andererseits die diagnostische Qualität nachhaltig gesichert. Auch die DEGUM-Zertifizierung dient letztlich der Qualitätssicherung von Ultraschallkursen durch die Einhaltung definierter Standards, darunter ein standardisiertes Curriculum, ein Mindestanteil von 50 % praktischer Kursinhalte und der Einsatz qualifizierter Lehrender.

Vor diesem Hintergrund verlagerte sich der Fokus von der inhaltlichen Gestaltung des Ausbildungskonzepts hin zu Fragen der methodischen Umsetzung. Insbesondere Lehrformate – ob Präsenz, digital oder hybrid – sowie die Ausgestaltung von Kompetenzüberprüfungen sind bislang heterogen gelöst. In der Gestaltung moderner Lehrformate müssen didaktische Qualität, Praxisrelevanz, Vereinbarkeit mit dem klinischen Alltag sowie die Rücksichtnahme auf individuelle Lernrealitäten gleichermaßen Beachtung finden. Erste Studien deuten darauf hin, dass Webinare in der theoretischen und auch praktischen Ultraschalllehre eine vergleichbare Effektivität wie klassische Präsenzformate aufweisen – ein Aspekt, der angesichts wachsender Flexibilitätsansprüche zunehmend an Bedeutung gewinnt [2–4].

Prüfungen sind seit jeher ein integraler Bestandteil medizinischer Ausbildung, da sie nicht nur der Leistungskontrolle dienen, sondern nachweislich einen positiven Einfluss auf den Lernerfolg haben [5]. Ihre konkrete Umsetzung ist somit nicht nur organisatorisch, sondern auch didaktisch bedeutsam und aus der medizinischen Lehre nicht wegzudenken.

## Material und Methoden

Die Datenerhebung erfolgte im Rahmen eines zweistufigen Verfahrens, beginnend mit einer Bestandsaufnahme vorhandener Kursangebote sowie anschließenden leitfadengestützten Interviews mit offenen Fragen an die Kursverantwortlichen.

### Bestandsaufnahme

Bei der Bestandsaufnahme der für die Fragestellung relevanten Ultraschallkurse in Deutschland wurden verfügbare Kurse mit dem Themengebiet „Kopf-Hals“ und „Schilddrüse“ berücksichtigt, wobei Kurse mit primärer Ausrichtung auf andere Fachgebiete ausgeschlossen wurden, sofern die genannten Themengebiete lediglich eine untergeordnete inhaltliche Gewichtung im Gesamtkursinhalt aufwiesen. Eingeschlossen wurden Fortbildungsangebote auf den Webseiten der vermeintlich am ehesten mit der Thematik befassten Fachgesellschaften (DEGUM, mit der für die Fachbereiche zuständigen Kopf-Hals-Sektion, Deutsche Gesellschaft für Hals-Nasen-Ohren-Heilkunde, Kopf- und Hals-Chirurgie [DGHNO-KHC] als Dachgesellschaft der anwendenden, klinisch tätigen HNO-Ärzte, und Deutsche Gesellschaft für Mund‑, Kiefer- und Gesichtschirurgie [DGMKG] als Gesamtverband aller Fachärzte für Mund‑, Kiefer- und Gesichtschirurgie). Darüber hinaus in die Auswertung eingeschlossen wurden klinikgebundene Weiterbildungen sowie Kursangebote, die im Rahmen einer systematischen Recherche mittels internetbasierter Suchmaschinen im Zeitraum von März bis Juli 2025 ermittelt wurden. Zu den meisten Kursen waren online detaillierte Flyer verfügbar, aus welchen zahlreiche relevante Angaben zu Struktur, Dauer, Inhalten, Lehrformaten und teilweise auch Prüfungsmodalitäten entnommen werden konnten. Diese wurden gründlich erfasst und vergleichend aufbereitet.

Im Anschluss daran wurden Telefoninterviews mit den jeweils benannten Kursverantwortlichen geführt. Wurden im Flyer oder auf der Kurswebsite mehrere Personen genannt, erfolgte die Kontaktaufnahme zur Terminierung des Interviews via Mail mit der fachlich hauptverantwortlichen Person.

### Ablauf des Telefoninterviews

Nach einer kurzen Vorstellung des Projekts wurde das mündliche Einverständnis zur Nutzung der Angaben eingeholt. Ziel war es, die recherchierten Informationen zu bestätigen sowie vertiefende Einblicke in die Kursgestaltung und insbesondere das Prüfungsformat zu gewinnen. Der Interviewleitfaden umfasste offene Fragen zur Zielgruppe, maximaler Teilnehmendenanzahl sowie dem Lehrformat mit der Nennung möglicher Vor- und Nachteile des Konzepts. Zudem wurden die Kursverantwortlichen gebeten Herausforderungen der Kursplanung, den Weiterentwicklungsbedarf und die aktuelle und künftige Rolle digitaler Formate zu benennen und einzuschätzen. Abschließend wurden Fragen zur Durchführung und Begründung von Leistungsüberprüfungen (theoretisch und/oder praktisch, digital und/oder in Präsenz) und zur Qualitätssicherung (Evaluation, Feedback, externe Kontrolle) gestellt. Raum für offene Ergänzungen schloss jedes Gespräch ab.

Die erhobenen Daten wurden quantitativ und qualitativ ausgewertet und systematisch zusammengefasst.

## Ergebnisse

Die Recherche ergab im angegebenen Zeitraum 9 Kursanbieter mit dem Schwerpunkt „Kopf-Hals-Sonographie“ und 4 Kursanbieter mit dem Schwerpunkt „Schilddrüse“. Eine Übersicht liefert Tab. 1. Da am Standort Erlangen 2 inhaltlich getrennte Kurse angeboten werden, die in Tab. 1 unter demselben Standort zusammengefasst sind, umfasst die tabellarische Darstellung 12 Einträge bei insgesamt 13 identifizierten Kursangeboten. Die theoretischen Themengebiete unterscheiden sich nicht wesentlich und sollen hier daher nur am Rande erwähnt werden. Im Grundkurs werden überwiegend physikalisch-technische Grundlagen, Sonoanatomie, Untersuchungstechniken, Indikationsstellung und Befunddokumentation vermittelt. Teilweise wird auch auf häufige Pathologien eingegangen. Im Aufbau- und Abschlusskurs sind die Themenblöcke darauf aufbauend spezialisierter, mit einem Schwerpunkt auf Pathologien, sonographischen Interventionen und Interdisziplinarität.

**Tab. 1:** Übersicht der identifizierten Kopf-Hals- und Schilddrüsen-Ultraschallkurse

Kursanbieter Ort	Kursleitung	Module	Turnus	Format	Leistungskontrolle	Evaluation	DEGUM-Zert.	Kurs existiert seit
UK Dresden, HNO	Prof. Dr. T. Beleites, Dr. M. Kemper	Grund‑, Aufbau- und Abschlusskurs	Jährliche Kursserie	Präsenz	Theoretisch mit Videos im Auditorium und OSCE	Standardisierter analoger Fragebogen	Ja	1993
UK Erlangen, HNO	Prof. Dr. S. Müller, PD Dr. M. Sievert, Prof. Dr. M. Koch	Grund‑, Aufbau- und Abschlusskurs, Schilddrüse I und II	Jährliche Kursserie	Präsenz	MCF anonym mit TED	Standardisierter analoger Fragebogen	Ja	1991
UK Frankfurt, MKG	Prof. Dr. Dr. R. Sader, Prof. Dr. Dr. B. Al-Nawas	Grund‑, Aufbau- und Abschlusskurs, Refresherkurs	Jährliche Kursserie	Präsenz	Persönliches Prüfungsgespräch mit praktischem Anteil	Standardisierte Evaluationsbögen der Weiterbildungsakademie MKG	Ja	1995
UK Freiburg, HNO	Prof. Dr. A. Knopf, Prof. Dr. Dr. R. Schmelzeisen, PD Dr. N. Mansour	Grund- Aufbau- und Abschlusskurs	Jährliche Kursserie	Präsenz	MCF anonym mit TED	Keine	Ja	2021
SonoForKlinik Mainz, Regensburg, Homburg	Prof. Dr. J. Künzel, Dr. J. Weimer	Grund‑, Aufbau- und Abschlusskurs	Mehrfach jährliche Kursserie	Hybrid	MCF und DOPS	Standardisierter digitaler Fragebogen	Ja	2020
Klinikum rechts der Isar, HNO München	Dr. Z. Zhu, Prof. Dr. B. Hofauer	Grund‑, Aufbau- und Abschlusskurs	Jährliche Kursserie	Präsenz	MCF anonym mit TED	Keine	Ja	Seit 41 Jahren mit Pausen
Stiftungsklinikum PROSELIS gGmbH, Standort Prosper-Hospital Recklinghausen	Prof. Dr. M. Teschner	Grund‑, Aufbau- und Abschlusskurs	Jährlich ein Modul	Präsenz	MCF anonym, Besprechung im Auditorium	Standardisierter analoger Fragebogen	Ja	2025
Bundeswehrkrankenhaus Ulm, HNO	Prof. Dr. Dr. h. c. M. Tisch, Prof. Dr. C. Offergeld	Grund‑, Aufbau- und Abschlusskurs	Jährliche Kursserie	Präsenz/Hybrid*	MCF und OSCE	Standardisierter analoger Fragebogen	Ja	1994
Im Rahmen des HNO-Kongresses der Deutschen Fortbildungsgesellschaft der HNO-Ärzte mbH, Mannheim	Prof. Dr. J. Künzel, Prof. Dr. med B. Hofauer	Demonstrations- und Refresherkurs	Jährliche Kursserie	Präsenz	Keine	Standardisierter analoger Kongress-Fragebogen	Ja	Seit 2023 in der jetzigen Struktur
SONO2learn	Dr. M. Braunfels, Prof. Dr. H. Strunk	Schilddrüse und Nebenschilddrüse inkl. Halslymphdrüsen	Konstant online	Online	MCF	Digital: Standardisierter Evaluationsbogen der Bundesärztekammer und Persönlich	Nein	2018
Sonowissen Dresden, Frankfurt	Dr. J. Aland	Kompaktkurs Schilddrüsensonographie	Mehrmals jährlich	Präsenz	MCF anonym mit TED	Standardisierter analoger Fragebogen	Nein	2018
UK Regensburg, HNO, Endokrinologie und Nuklearmedizin	Prof. Dr. C. Bohr, Prof. Dr. D. Hellwig, Prof. Dr. M. Müller-Schilling, Prof. Dr. H.-J. Schlitt, Dr. S. Heidemanns, Prof. Dr. J. Grosse	Schilddrüse I und II	Jährlich	Präsenz	Praktisch: Kompetenzkriterien, die erreicht werden müssen und direktes Feedback	Standardisierter digitaler Fragebogen	Ja	2024

*DEGUM *Deutsche Gesellschaft für Ultraschall in der Medizin,* DOPS *„direct observation of procedural skills“,* MCF *Multiple-Choice-Fragen,* OSCE *„objective structured clinical examination“,* TED *Tele-Dialog-System (elektronisches Abstimmungssystem),* UK* Universitätsklinik, *Zert.* Zertifizierung

*Hybrider Kurs im Rahmen einer Studie

Weiterhin in Präsenz werden 10 von 13 Kursen (76,92 %) abgehalten. Einen Umfang von 2 Tagen haben 9 Kurse (69,23 %). Davon abzugrenzen ist der dreitägige Grundkurs in Frankfurt, der eintägige Refresherkurs und der eintägige Sonowissen-Schilddrüsenkompaktkurs (Akademie für kardiovaskulären Ultraschall, Dres. Aland/Jungblut GbR, Frankfurt am Main, Deutschland) sowie der SONO2learn-Online-Kurs (Fa. SONO2learn GmbH, Köln, Deutschland), dessen Videolehrmaterial 13 h umfasst und on demand über 3 Jahre beliebig häufig abgerufen werden kann. Das Frankfurter Kurssystem zeichnet sich zusätzlich durch ein einzigartiges Konzept aus, bei dem alle Kursteile zeitgleich stattfinden und die Teilnehmenden, ähnlich wie bei einem Kongress, von einer Vielzahl parallel angebotener Module profitieren können.

Die maximale Teilnehmendenanzahl variiert bis auf den Online-Kurs, bei welchem diese unbegrenzt ist. Mit Ausnahme des Refresherkurses werden bei allen Kursen, die praktische Übungen beinhalten, maximal 5 Teilnehmende pro Tutor eingeplant, was einem Maximum von 20–50 Teilnehmenden pro Kurs entspricht.

Für alle Kurse werden Fortbildungspunkte bei den jeweils zuständigen Landesärztekammern beantragt. Lernerfolgskontrollen werden bei 10 von 12 Anbietern (83 %) durchgeführt. Auf eine klassische Prüfung verzichten 2 Anbieter, wobei bei einem dieser beiden im praktischen Kursteil die Sicherstellung definierter Kompetenzkriterien durch gezielte Supervision vorgesehen ist. Die konkrete Ausgestaltung der Leistungsüberprüfung ist ebenfalls heterogen: Multiple-Choice-Formate setzen 8 Anbieter (62 %) ein, 3 (23 %) nutzen praktische Prüfungsformate wie OSCE (*n* = 2) oder DOPS (*n* = 1) in Kombination mit theoretischen Anteilen, und ein Anbieter (8 %) führt ein mündliches Format mit einem Prüfungsgespräch und einer nichtstandardisierten Überprüfung des Untersuchungsablaufs durch.

Reine Online-Kurse wie SONO2learn oder Kurse mit einem geringen Praxisanteil erfüllen § 6 Absatz 2 Buchstabe d) der Ultraschallvereinbarung nicht, der vorsieht, dass praktische Übungen die Hälfte der Gesamtkurszeit umfassen sollten [6]. In der geforderten Kombination erfüllen alle anderen Kurse die Voraussetzungen der KV für eine Abrechnungserlaubnis.

Die Bereitstellung von Lehrmaterial zur Nachbearbeitung des Lernstoffs hängt vom Vortragenden ab. Auf den vollständig digitalen Kurs *SONO2learn* haben Teilnehmende 3 Jahre lang Zugriff. SonoForKlinik (Website-Anbieter SonoForKlinik, Johannes Matthias Weimer, Waldbrunn, Deutschland) stellt im Rahmen des hybriden Kurssystems Lehrmaterialien mit der Möglichkeit zur digitalen Nachbearbeitung zur Verfügung.

Bezüglich der Kursgebühren wurden zwar deutliche Unterschiede zwischen den Anbietern festgestellt, auf die Angabe konkreter Kursgebühren wird jedoch bewusst verzichtet, da diese von zahlreichen Faktoren abhängen, wie etwa der Kursdauer, der Einbeziehung von Verpflegungsleistungen, möglichen Sponsorings sowie variablen Kosten der Teilnehmenden für Anreise und Unterkunft.

### Telefoninterview

Pro Kursanbieter wurde nach den aufgeführten Kriterien in 11 Fällen eine Person, in einem Fall 2 Personen interviewt. Da der individuelle Rückschluss auf die befragten Personen für die Auswertung nicht relevant ist, wurden die Angaben anonymisiert und sind im Folgenden überwiegend nicht mit einzelnen Personen in Verbindung zu bringen. Insgesamt konnten 11 Interviews vollständig ausgewertet werden. Ein weiteres Interview musste aus Zeitgründen vorzeitig beendet werden und liegt daher nur in Teilen vor.

### Zielgruppe

Nach Einschätzung der befragten Anbieter richten sich die Kurse grundsätzlich an alle Interessierten. Im Fall der meisten Kurse (*n* = 10) führt das zu einer überwiegenden Teilnahme von Assistenzärztinnen und -ärzten sowie Fachärztinnen und -ärzten aus dem HNO- und MKG-Fachbereich für die Kopf-Hals-Kurse. Bei den Schilddrüsenkursen (*n* = 3) wird eine interdisziplinäre Teilnahme angegeben, wobei ein Kursverantwortlicher zusätzlich von einem insgesamt höheren Anteil an Fachärztinnen und -ärzten berichtete.

### Wahl des Ausbildungsformats

Die Anbieter von Präsenzkursen nannten diverse Vorteile dieser Form der Durchführung, wie die Möglichkeit, individuelle Fragen direkt zu beantworten und durch den direkten Kontakt mit dem Auditorium ein besseres Gespür für das Verständnis der Teilnehmenden zu entwickeln. Im persönlichen Kontakt sei eine intensivere Interaktion möglich, wobei einschränkend ergänzt wurde, dass diese erfahrungsgemäß im praktischen Kursteil mit Abstand am höchsten sei. Als besondere Stärke des Präsenzformats wurde dessen Eignung zur Vermittlung praktischer Ultraschallfertigkeiten, etwa durch die wiederholte Durchführung standardisierter Untersuchungsabläufe, hervorgehoben. Zugleich wurde infrage gestellt, inwieweit sich praktische Übungen in ein Online-Format übertragen lassen. Daneben wurde die Überprüfung der Ultraschallpraxis als wichtiges Element genannt, wobei sich im Verlauf der Jahre wiederholt eine teilweise deutliche Diskrepanz zwischen Eigen- und Fremdeinschätzung der Lernenden zeigte. Zudem ließen sich die von den Teilnehmenden zwischen den Kursmodulen erhobenen Befunde im persönlichen Austausch zuverlässiger evaluieren. Ein Anbieter hob zwar den didaktischen Vorteil von Präsenzkursen hervor, erwog jedoch gleichzeitig – dem aktuellen Trend folgend –, künftig Teile der Vorträge vorab online bereitzustellen. Herausgehoben wurde außerdem der persönliche Kontakt mit einer anregenden Gesprächsatmosphäre, und zwar nicht nur während des Kurses, sondern auch während der Pausen und bei den teilweise integrierten Abendveranstaltungen. Als potenzieller Nachteil von Präsenzformaten wurde die eingeschränkte Flexibilität und Erreichbarkeit sowie Kosten und Aufwand durch die Anreise genannt.

Die Anbieter digitaler oder hybrider Kurse sahen als wesentliche Vorteile ihrer Formate die größere zeitliche und örtliche Flexibilität, die Möglichkeit eines individuelleren Lerntempos sowie die gezielte Vor- und Nachbereitung der Inhalte. Gut strukturierte digitale Angebote können theoretische Grundlagen systematisch und praxisnah vermitteln und so eine effizientere Nutzung der Präsenzzeit für praktische Übungen ermöglichen. Ein Anbieter hob ergänzend die didaktische Stringenz und fachliche Tiefe solcher Kurse hervor, die – im Gegensatz zu oft fragmentierten Lernsituationen im Klinikalltag – eine strukturierte Wissensvermittlung bis zu einem hohen fachlichen Niveau erlaubten. Als potenzielle Nachteile digitaler Formate wurden der eingeschränkte direkte Austausch und die Notwendigkeit eines hohen Maßes an Eigenverantwortung genannt, wobei Letzteres zugleich als Chance zur Förderung selbstgesteuerten Lernens bewertet wurde.

### Herausforderungen der Kursplanung und -durchführung

Als zentrale Herausforderung bei der Durchführung des Kurses wurde von fast allen Befragten (*n* = 10) die Probandenrekrutierung hervorgehoben. Auch zusätzliche organisatorische Anforderungen des praktischen Kursanteils – etwa die Sicherstellung ausreichender Geräte, die frühzeitige Reservierung geeigneter Räumlichkeiten sowie die Verfügbarkeit qualifizierter Referenten und Tutoren – stellten eine nicht zu unterschätzende Herausforderung dar. Besonders relevant ist hierbei der Zeitfaktor: Zum einen müssen Referenten und Tutoren von ihren regulären klinischen Aufgaben freigestellt werden, zum anderen erfordert auch die Organisation der Kurse selbst neben der regulären Vollzeittätigkeit einen erheblichen zeitlichen Mehraufwand. Die Rekrutierung, insbesondere von erfahrenen Fachärztinnen und Fachärzten, die trotz Interesse nicht aktiv nach Fortbildungsangeboten suchen, wurde ebenfalls angeführt und dabei die Bedeutung einer gezielten Ansprache zur Vermittlung der Wissensauffrischung und des praxisrelevanten Mehrwerts eines Kurses hervorgehoben.

Auf der Seite der Teilnehmenden teilte ein Anbieter die Erkenntnis seiner Kursevaluationen, die in den letzten Jahren zeigte, dass auch viele Teilnehmende die Organisation ihrer Abwesenheit von den eigenen Kliniken als Herausforderung empfinden.

### Verpflichtender Ultraschallkurs im Rahmen der Facharztweiterbildung

Gegen die Einführung eines verpflichtenden Ultraschallkurses in der Facharztweiterbildung sprachen sich 8 Kursverantwortliche aus und verwiesen auf den höheren Lernerfolg bei freiwilliger Teilnahme sowie die Vermeidung zusätzlicher Regularien. Zusätzlich betonte ein Anbieter die wünschenswerte, jedoch organisatorisch schwer realisierbare Beteiligung erfahrener DEGUM-Kursleitender an jeder Facharztweiterbildung. Eine Annäherung an dieses Ziel könnte darin bestehen, allen Weiterbildungsassistentinnen und -assistenten im Rahmen ihrer Ausbildung auf freiwilliger Basis die Teilnahme an einem UK-KH zu ermöglichen.

Befürwortend argumentierten 3 Interviewte mit der zentralen Bedeutung der Sonographie für Klinik und Praxis und dem Potenzial, durch einen strukturierten, verpflichtenden Kurs einen einheitlichen Mindeststandard zu sichern und systematisch das Niveau der Anwendung insgesamt zu heben. Eine weitere Anbieterin sprach sich, in Abhängigkeit von der Fachrichtung und den Zielorganen, für eine Verpflichtung aus, da ihrer Erfahrung nach an Kliniken teilweise keine ausreichende Ausbildungsintensität- und -kapazität gewährleistet ist.

### Zukunft der Ultraschallausbildung

Im Hinblick auf die künftige Entwicklung der Ultraschallausbildung im Schwerpunkt Kopf-Hals-Bereich und Schilddrüse hoben alle kontaktierten Anbieter die zunehmende Bedeutung einer fundierten diagnostischen Ausbildung hervor. Explizit wurde die Rolle der Sonographie in der chirurgischen Ausbildung von 3 Anbietern unterstrichen, da ihrer Erfahrung nach durch ein verbessertes anatomisches Verständnis auch eine höhere Qualität der Diagnostik und operativen Eingriffe erreicht werden kann. Ein Kursorganisator propagierte die zunehmende Relevanz der interventionellen Sonographie, die sein Team zur Entwicklung eines haptischen Lehrmodells motivierte, welches an anderer Stelle bereits veröffentlicht wurde [7]. Neben der gesteigerten diagnostischen Sicherheit durch fundierte Ultraschallkompetenz werde letztlich die therapeutische Entscheidung beschleunigt, was direkt patientenrelevant sei.

Ein Anbieter erachtete eine regelhafte Erweiterung des UK-KH um die Schilddrüsendiagnostik als sinnvoll, die 2026 Einzug in sein Kurskonzept finden wird. Ferner begrüßte ein anderer Anbieter das inzwischen weit verbreitete Verständnis dafür, dass hochqualitative sonographische Abklärungen der Schilddrüse möglich sind und interdisziplinäre Kurse sowohl aktuell als auch künftig dazu beitragen können, Überdiagnostik (bspw. Szintigraphien) zu vermeiden.

In einem Interview wurden jedoch auch zeitliche und finanzielle Herausforderungen benannt, insbesondere durch eingeschränkte Ausbildungsressourcen und die hohen Kosten hochwertiger Geräte. Wenngleich die Einschränkungen an der eigenen Ausbildungseinrichtung als überschaubar eingeschätzt wurden, betonte der Interviewte, dass die gestiegene Arbeitsdichte die qualifizierte Weiterbildung erschwere, da verfügbare Zeitressourcen zunehmend mit wertschöpfenden Tätigkeiten belegt würden und somit weniger Zeit für Ausbildung bleibe.

Mit Blick auf die Chancen technologischer Entwicklungen verwiesen 2 Anbieter auf das Spannungsfeld zwischen ärztlicher Expertise und mittels Künstlicher Intelligenz (KI-)gestützter Bildanalyse: Während KI durch automatische Bildvermessung oder Mustererkennung die ärztliche Arbeit erheblich entlasten könne, bleibe die ärztliche Entscheidungskompetenz unverzichtbar. Setzt man sich aktiv mit Möglichkeiten und Grenzen der KI auch in der Lehre auseinander, könne durch eine Symbiose ein besonderer Mehrwert für Patientinnen und Patienten entstehen.

Eine Kursverantwortliche betonte die zukünftige Notwendigkeit einer klaren Qualitätsfokussierung, etwa durch eine Trennung der Ultraschallqualifikation von der Weiterbildung zum Facharzt. In einem anderen Interview wurde hervorgehoben, dass Qualitätssicherung nicht nur die initiale Ausbildung umfassen, sondern über den gesamten Karriereverlauf hinweg verankert werden sollte. Dies erscheine insbesondere deshalb bedeutsam, da die Sonographie v. a. im niedergelassenen Bereich oftmals unzureichend vergütet würde, wodurch die Motivation zur regelmäßigen Anwendung sinke, was die Untersuchungsqualität langfristig beeinträchtigen könne.

### Rolle digitaler oder hybrider Lernformate

Alle befragten Anbieter erwarten, dass digitale und hybride Lernformate künftig eine zentrale Rolle in der Ultraschallausbildung einnehmen werden. So wurde in den Interviews das erhebliche Entwicklungspotenzial in der Ultraschalllehre hervorgehoben, das u. a. durch die systematische Weitergabe klinischer Expertise in kompakter und didaktisch strukturierter Form an die nächste Generation adressiert werden könne. Vorteil eines hybriden Formats sei die Möglichkeit einer visuell ansprechenden Aufbereitung komplexer Themen sowie eine gleichbleibend hohe didaktische und inhaltliche Qualität, unabhängig von der Verfügbarkeit einzelner Ausbilder, was die Flexibilität erhöhe und damit die Integration theoretischer Inhalte in den Arbeitsalltag ermögliche. Vor dem Hintergrund gesellschaftlicher Digitalisierung und der zunehmenden Anzahl von „digital natives“ unter den Teilnehmenden wachse ohnehin die Akzeptanz digitaler Formate. So befand sich ein Anbieter zum Zeitpunkt des Interviews in Vorbereitung einer Kooperation mit *123Sono* (Fa. 123 Sonography GmbH, Wien, Österreich) zur Integration digitaler Kursanteile.

Im Gegensatz dazu plädierte ein Befrager dafür, digitale Formate primär als Repetitorien zur Vorbereitung auf die Facharztprüfung einzusetzen, während ein weiterer ihren Nutzen v. a. im „Prelearning“ zur effizienteren Kursvorbereitung sah. Zudem hoben 2 Anbieter hervor, dass trotz des wachsenden Angebots digitaler Lernmöglichkeiten die Präsenzzeit nicht ausschließlich mit praktischen Übungen gefüllt werden sollte, um eine abwechslungsreiche Kursgestaltung zu gewährleisten.

Zuletzt gab ein Kursverantwortlicher zu bedenken, dass ein höheres Maß an Eigenverantwortung der Teilnehmenden durch digitale Kursanteile mit einer verstärkten Kontrolle der Lerninhalte, beispielsweise in Form von Prüfungen, abgesichert werden müsse, um einen Qualitätsverlust zu vermeiden.

### Prüfungsformat

Am häufigsten kamen Multiple-Choice-Fragen (MCF) zum Einsatz, teils in analoger oder digitaler Form, teils in Kombination mit interaktiven TED-Systemen („audience response systems“) und ergänzt durch bild- und videobasierte Fragen (*n* = 8). Dabei wurde betont, dass diese Formate eine standardisierte Erfassung theoretischen Wissens ermöglichen und durch die Einbindung visueller Elemente zusätzlich das diagnostische Denken fördern könnten. Darüber hinaus oder als alleiniges Format integrierten 4 Anbieter praktische Prüfungsanteile, beispielsweise in Form eines persönlichen Prüfungsgesprächs mit praktischer Demonstration, einer „direct observation of procedural skills“ (DOPS) oder einer „objective structured clinical examination“ (OSCE). Anhand eines Prüfungsbogens werden die relevanten Fertigkeiten systematisch erhoben und überprüft. Die Anbieter argumentierten einerseits, dass dieses Prüfungsformat den Anforderungen des klinischen Alltags besonders gut entspreche, und andererseits, dass sich praktische Fähigkeiten, einschließlich der Ultraschalluntersuchung, damit valide und nachvollziehbar bewerten lassen.

Im Interview wurde zusätzlich angemerkt, dass praktische Prüfungen auch für die Lehrenden eine wertvolle Rückmeldung darstellen können, da sich wiederholende Fehler mehrerer Teilnehmender als Hinweis auf missverstandene Inhalte und damit als Ansatzpunkt für die Optimierung der Lehre verstehen lassen. Eine andere Interviewte konzentrierte sich bei der praktischen Lernerfolgskontrolle auf unmittelbares Feedback an die Teilnehmenden und das Erreichen klar definierter „checkpoints“ zur Überprüfung des Ausbildungsziels, wodurch der kontinuierliche Lernprozess gezielt unterstützt werden soll. Auch das Prüfungsformat eines weiteren Anbieters setzte auf unmittelbare Rückmeldung, jedoch in digitaler Form: Die Teilnehmenden erhalten direkt nach der Beantwortung der MC-Fragen am Bildschirm eine Rückmeldung, ob ihre Auswahl korrekt war. Zusätzlich werden ausführliche Begründungen angezeigt, die die Richtigkeit oder Fehlerhaftigkeit der Antwort erläutern und so das Verständnis vertiefen und den nachhaltigen Lerneffekt fördern sollen.

## Diskussion

Die Ergebnisse zeigen ein heterogenes Feld der Lehrformate, wobei Präsenzformate weiterhin überwiegen. Im Gegensatz hierzu zeigt sich eine weitgehende inhaltliche Übereinstimmung der Kurse. Mögliche Gründe hierfür könnten in der Ausrichtung auf eine ähnliche Zielgruppe sowie in den normierenden Vorgaben durch die KV und die DEGUM liegen. Diese Homogenisierung bietet zweifellos Chancen, etwa im Hinblick auf eine gute Vergleichbarkeit der Kursinhalte und eine flächendeckende Qualitätssicherung – ein zentrales Ziel der Ultraschallvereinbarung [5]. Gleichzeitig birgt der damit einhergehende geringe Handlungsspielraum das Risiko, den didaktischen Gestaltungsspielraum einzuschränken, insbesondere mit Blick auf innovative digitale Lehrformate wie interaktive Online-Module, asynchrone Lerneinheiten oder virtuelle Live-Demonstrationen.

Alle befragten Lehrenden gaben in den Interviews an, dass digitalen Lehrinhalten in Zukunft eine wachsende Bedeutung zukommen werde – auch wenn konkrete Umsetzungskonzepte nicht benannt wurden. Erste Studien liefern Hinweise darauf, dass moderne Lehrformate bei der Vermittlung praktischer Fertigkeiten vergleichbare Ergebnisse erzielen können wie klassische Präsenzformate [2, 8]. Einige dieser Studien sind im Zuge der COVID-19-Pandemie-Situation entstanden und unterlagen somit speziellen Rahmenbedingungen. Gleichwohl reflektieren diese Arbeiten aber auch den methodischen Ansporn und die Dringlichkeit zur Lösung von Problemen, welche anscheinend nur unter den spezifischen Rahmenbedingungen einer Pandemie akzeptabel zu sein scheinen. Um die Qualität solcher Ansätze valide bewerten zu können, sind jedoch weitere methodisch weiterführende Untersuchungen erforderlich, gepaart mit einer grundsätzlichen Offenheit für innovative Ansätze.

Eine mögliche Perspektive wäre, zunächst standardisierte Instrumente zur objektiven Erfassung der Untersuchungsqualität nach Abschluss eines Ultraschallkurses zu etablieren. Auf dieser Grundlage ließen sich nicht nur bestehende Formate evaluieren, sondern auch potenzielle Innovationen gezielt hinsichtlich ihres Outcomes vergleichen. Es wäre wünschenswert, die daraus gewonnenen Erkenntnisse perspektivisch in die curricularen Vorgaben und Zertifizierungsstandards einfließen zu lassen, um den hohen Qualitätsansprüchen der Ultraschalldiagnostik auch im Wandel der Zeit gerecht zu werden.

Ein Ansatz besteht in der überregional einheitlichen Erhebung teilnehmerbasierter Evaluationen, die als subjektives Instrument Rückschlüsse auf die wahrgenommene Qualität und Relevanz der Kursformate ermöglichen könnte. Die vorliegenden Ergebnisse zeigen, dass dies bislang nicht konsequent umgesetzt wird und Evaluationsformate erheblich variieren.

Ein objektiver Ansatz, den Kompetenzerwerb systematisch zu erfassen, ist die gezielte Gestaltung von Prüfungsformaten. Dass sich die Lehrenden im Rahmen der Ultraschallausbildung bereits aktiv mit dieser Frage auseinandersetzen, spiegelt sich in der Bandbreite der eingesetzten Verfahren wider. Diese Vielfalt deckt sich mit den Ergebnissen der systematischen Übersichtsarbeit von Höhne et al., die verschiedene etablierte Prüfungsformate beschreibt, ohne jedoch eine eindeutige Empfehlung für ein optimales Vorgehen geben zu können [9]. Weit verbreitet in der medizinischen Ausbildung i. Allg. und innerhalb der untersuchten Kurse sind MC-Fragen, die reines Faktenwissen abfragen und in analoger Form als Papierfragebogen oder digital über elektronische Abstimmungssysteme (TED) gestellt werden. Angesichts leistungsfähiger KI-Systeme wie ChatGPT oder Google Gemini ist die Aussagekraft dieses Prüfungsformats zur Erfassung theoretischen Wissens kritisch zu betrachten. Lehrende sind daher gefordert, bestehende Formate weiterzuentwickeln und stärker auf prüfungsdidaktische Konzepte zu setzen, die Verständnis, Anwendungskompetenz und klinisches Denken in den Mittelpunkt stellen.

Dass dieser Wandel bereits begonnen hat, zeigen neben bild- und videobasierten MC-Fragen auch die eingesetzten praxisnahen Prüfungsformate, wie die OSCE oder die DOPS. Beide gelten in der medizinischen Ausbildung als effektive Verfahren zur Beurteilung klinisch-praktischer Fähigkeiten und werden zusätzlich von den Teilnehmenden als besonders lernförderlich wahrgenommen [10, 11]. Rink et al. publizierten erst kürzlich ihr angewendetes und evaluiertes DOPS-Konzept für Kopf-Hals-Ultraschallkurse [12]. Die Autoren bestätigten ebenfalls die hohe Akzeptanz dieses Prüfungsformats, betonten jedoch zugleich, dass eine umfassende Validierung noch aussteht. Anhand dieser Beispiele soll verdeutlicht werden, dass Prüfungen nicht nur der Lernerfolgskontrolle dienen, sondern auch den Lernprozess strukturieren, Lernziele sichtbar machen und nachhaltige Kompetenzentwicklung fördern. Darüber hinaus ermöglichen sie, sofern didaktisch sinnvoll konzipiert und gezielt eingesetzt, individuelles und differenziertes Feedback, das die Lernenden in ihrer Weiterentwicklung gezielt unterstützt.

Wie bereits angeführt, gibt es kein „gut“ oder „schlecht“, sondern eine Vielfalt an Prüfungsmöglichkeiten mit unterschiedlichem Fokus, welche – insgesamt gesehen – das Lernverhalten steuern können. Es scheint sich bereits jetzt abzuzeichnen, dass sich zukünftig die Tendenz zu weniger Faktenwissen und dafür mehr Handlungs- und Begründungswissen etablieren wird. Eine konstruktive Angleichung („constructive alignment“) in der Kopf-Hals-Sonographie wäre so gesehen nicht nur innovativ, sondern eine qualitätsfördernde Maßnahme im Sinne der aktuellen „Stakeholder“ sowie eine Premiere. Die Medizindidaktik kann hier beratend wertvolle Hilfe leisten, wenn es denn von den Verantwortlichen und „Gatekeepern“ angenommen wird. Eine koordinierte Prüfungslösung für die aufgeführten zertifizierten Kurse könnte hier einen Anfang darstellen.

### Limitationen

Ein potenzieller „Bias“ ergibt sich daraus, dass alle Interviewpartner selbst Kurse anbieten und somit zu einer positiven Selbstdarstellung neigen könnten. Auch die Autorin und die Autoren sind in der Ultraschalllehre tätig, was die Objektivität beeinflussen kann.

Die Wahl eines leitfadengestützten Interviews mit offenen Fragen stellt eine methodische Einschränkung dar, wurde jedoch bewusst gewählt, da das Ziel der Arbeit die Erstellung einer Übersicht der aktuell verfügbaren Lehrformate und Prüfungsstrukturen im Bereich des Kopf-Hals-Ultraschalls war. Die konzeptionelle Heterogenität der Kurse hätte sich mit standardisierten quantitativen Erhebungsinstrumenten nur unzureichend erfassen lassen. Zudem stehen keine validierten objektiven Messinstrumente zur Bewertung curricular übergreifender Lehrqualität und praktischer Ultraschallkompetenz zur Verfügung. Die offene Erhebung ermöglichte die differenzierte Darstellung individueller Begründungen der Kursverantwortlichen, unterliegt jedoch einer interpretativen Verdichtung, wodurch nicht alle Nuancen und inhaltlichen Tiefen der Aussagen vollständig abgebildet werden können.

Zudem handelt es sich um eine Momentaufnahme einer dynamischen Kurslandschaft, in der bereits kurz nach Abschluss der Erhebung neue Kurse angekündigt wurden, etwa an der Universitätsklinik Magdeburg und von der Akademie für medizinische Fortbildung der Ärztekammer Westfalen-Lippe. Zuletzt ist nicht auszuschließen, dass kleinere oder nicht öffentlich beworbene Kurse in der Recherche unberücksichtigt geblieben sind.

## Fazit für die Praxis

- Kopf-Hals-Ultraschallkurse in Deutschland weisen eine deutliche methodische Vielfalt auf.
- Die eingesetzten Prüfungsformate variieren erheblich.
- Standardisierte, kompetenzorientierte, validierte Prüfverfahren fehlen bislang, könnten die Ausbildungsqualität künftig jedoch verbessern.

## Data Availability

Die erhobenen Datensätze können auf begründete Anfrage in anonymisierter Form beim korrespondierenden Autor angefordert werden. Die Daten befinden sich auf einem Datenspeicher am Bundeswehrkrankenhaus Ulm.
